# Chronic opioid use is associated with higher antibody response to influenza vaccination in people living with HIV

**DOI:** 10.3389/fimmu.2025.1686103

**Published:** 2025-12-17

**Authors:** Christine M. Dang, C. Mindy Nelson, Rajendra N. Pahwa, Hansel E. Tookes, Daniel J. Feaster, Prabhsimran Singh, Allan E. Rodriguez, David W. Forrest, Nobuyo Nakamura, Priya P. Ghanta, Dushyantha T. Jayaweera, Akshay Iyer, Suresh Pallikkuth, Savita G. Pahwa

**Affiliations:** 1 Department of Microbiology and Immunology, University of Miami, Miller School of Medicine, Miami, FL, United States; 2 Department of Public Health Sciences, University of Miami, Miller School of Medicine, Miami, FL, United States; 3 Department of Medicine, Division of Infectious Diseases, University of Miami, Miller School of Medicine, Miami, FL, United States

**Keywords:** HIV and opioids, immunity in OUD, vaccine and OUD, HIV and OUD, HIV and OUD immunity

## Abstract

**Introduction:**

We previously reported that opioid use disorder (OUD) heightens inflammation in people with HIV (PWH). Underlying inflammation is considered to be detrimental to immune responses to influenza vaccine in PWH. Here, we tested the hypothesis that influenza vaccine responses in PWH with OUD would have a greater compromise than PWH without OUD or in people without HIV (PWoH) with OUD.

**Methods:**

We enrolled 244 participants based on OUD (OP+/-) and HIV status (HIV+/-) into an influenza vaccine study in which we analyzed hemagglutination inhibition (HAI) titer at pre- (T0) and approximately 7 days (T1), 4 weeks (T2), and 6 months (T3) post-vaccination with a seasonal quadrivalent influenza vaccine. The log2 of T2/T0 fold change (FC) for each of the 4 antigens H1N1, H3N2, B1- Victoria, B2-Yamagata and whole vaccine was calculated. Regression modeling was conducted to assess effects of HIV and opioids and other covariates on antibody response.

**Results:**

All participant groups (39 HIV+OP+, 66 HIV-OP+, 67 HIV+OP- and 71 HIV-OP-) demonstrated increases in HAI titer from T0 to T2 for all 4 antigens and whole vaccine with the HIV+OP- group manifesting the lowest HAI titers. The T2/T0 FC for H3N2, B1, B2, and whole vaccine was greater in both OP+ groups (PWH and PWoH) than in HIV+OP-, and was similar to that in HIV-OP- control group. Significant interactions between HIV+ status and opioid use were observed after controlling for demographics, previous influenza vaccine history, baseline titers, and other substance use.

**Discussion:**

Interaction between HIV and opioid use on immune function results in enhanced antibody response to influenza vaccination in PWH.

## Background

The opioid crisis with 3.7 million people in the US injecting drugs (1) significantly intersects with the HIV epidemic, posing unique challenges for individuals and public health systems. Opioid use can increase the risk of HIV transmission, exacerbate disease progression, and complicate goals of ending the HIV Epidemic. People with HIV (PWH) experience chronic pain 25% to 90% more than people without HIV (PWoH), leading to a greater likelihood of being prescribed opioids and an increased risk of developing an opioid use disorder (OUD) (2). Opioids are prescribed more frequently to PWH, with estimates indicating that the proportion receiving opioid prescriptions ranges from 21% to 53% (2). In a recent study, the prevalence of past-year non-medical opioid use was 11.1% among PWH, compared to 4.2% among those without HIV (3). Recent findings from our laboratory indicate heightened inflammation and immune activation among PWH with OUD (4). We have previously reported immune dysfunction in virally suppressed PWH, characterized by a poor immune response to seasonal influenza vaccination, attributed to higher immune activation, inflammation, and deficiencies in the quality and quantity of immune cells involved in antibody responses (5–7). However, the impact of opioids on the immune system of PWH is not well characterized, and there is ongoing debate regarding whether opioids exert immunosuppressive or immunostimulatory effects (8).

Hospitalization due to infectious diseases and bacterial infections is more frequent among people who inject drugs (PWID) (9, 10). PWID and people who use drugs are more prone to poor outcomes such as longer hospital stays and higher rates of intensive care unit (ICU) readmission (10, 11). Over the last decade, there has been an increasing trend of injection drug use, which correlated with the outbreaks of hepatitis C, hepatitis B, and increased risk of acquiring HIV infection (12). Vaccinations are recommended for PWH and people who use drugs to reduce the risk of serious infections (11). In the United States, routine influenza vaccinations are recommended for all persons aged ≥ 6 months (13). Between 2010-2022, it is estimated that influenza resulted in 100,000 – 710,000 hospitalizations and 4,900 – 52,000 deaths annually (14). Antibody response to influenza vaccination in the context of chronic opioid use and HIV is not well understood. To address this concern, OPIS (OPioid Immunity Study) was designed to investigate the effect of OUD on influenza vaccine-induced antibody responses in people with and without HIV. We tested the hypothesis that PWH plus OUD would have a greater compromise in influenza vaccine responses than PWH without OUD or in PWoH with OUD. Contrary to our hypothesis, this study revealed provocative findings of higher influenza vaccine responses in PWH with OUD compared to non-opioid using PWH confirming prior preliminary observations (15).

## Materials and methods

### Study design

**Figure 1:**
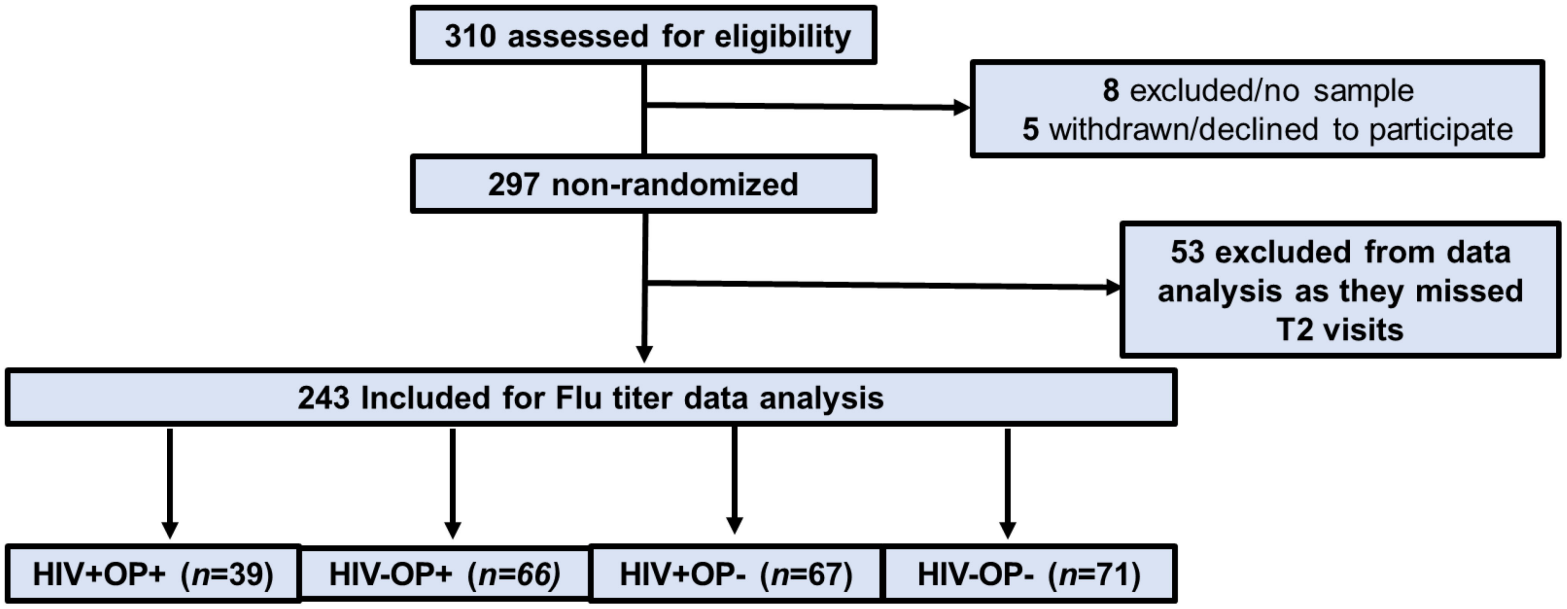
OPIS study enrollment information. Flowchart of participants enrolled and tested for flu titer analysis in the OPIS study.

Between September 2020 – May 2023, a total of 310 participants were screened at the University of Miami for OUD and HIV status in the Infectious Disease Elimination Act (IDEA) clinic, the general Infectious Diseases Clinic and through community outreach for the OPIS study (Clin trial # NCT04304768) (**Figure 1**). Additional details on this cohort were first described in (4). Urine samples were collected in 14-drug urine drug screen (UDS) (12panelnow.com) cups in all participants (4). Inclusion criteria for OUD included self-reported opioid use by injection or prescription for 90 or more days and a positive opioid test on the baseline (T0) Urine Drug Screen (UDS). 297 were randomized into the study of which 53 participants missed the day 28 (T2) study visit. The remaining 243 participants from four study groups: group 1, (HIV+OP+, n=39); group 2, (HIV-OP+, n=66); group 3, (HIV+OP-, n=67); and group 4, (HIV-OP-, n=71) underwent data analysis. All PWH were on antiretroviral therapy (ART) for at least six months, with most participants having plasma HIV RNA levels below 200 copies/mL, with IQR 20–332 copies/mL for HIV+OP+ and 20–132 for HIV+OP-. Qualitative UDS information specific to this cohort is listed in **Supplementary Table S1**. Fentanyl (44%) and buprenorphine (46%) use were common in HIV+OP+ while fentanyl was the predominant (80%) drug in HIV-OP+. The study period spanned three influenza seasons during which participants were enrolled for a period of 12 months during one of the three influenza seasons. Participants received a single dose of non-adjuvanted standard seasonal influenza vaccination during the specific season in which they were enrolled. Peripheral venous blood was collected in serum separation tubes for serum separation and in heparin containing tubes for peripheral blood mononuclear cell (PBMC) isolation at pre-vaccination (T0), approximately day 7-post-vaccination (T1), approximately day 21-28- post-vaccination (T2), and approximately 6 months post-vaccination (T3) (**Supplementary Figure S1**). Serum aliquots were frozen at -80°C. PBMC were cryopreserved in liquid nitrogen until use. Demographic information and clinical data based on self-reported questionnaires were stored in a REDCap database at each visit.

### Hemagglutination inhibition assay for antibody response to vaccination

Frozen serum samples were thawed and tested for antibody titers against seasonal influenza antigens (H1N1, H3N2, B1 Victoria, B2 Yamagata) and whole vaccine (WV). Seasonal influenza antigens were generously provided by the Center for Biologics Evaluation and Research (CBER). The following antigens were tested: (1) Season 2020-2021: A/Nebraska/14/2019 (an A/Hawaii/70/2019 (H1N1)pdm09-like virus); A/Hong Kong/2671/2019 IVR-208 (an A/HongKong/2671/2019 (H3N2)-like virus); B/Darwin/7/2019 (a B/Washington/02/2019-like virus); B/Singapore/INFTT-16-0610/2016 (a B/Phuket/3073/2013-like virus); (2) 2021-2022: A/Victoria/2570/2019 (H1N1) pdm09-like virus; A/Tasmania/503/2020 IVR-221 (H3N2); B/Washington/02/2019- like virus (B/Victoria lineage); B/Phuket/3073/2013-like virus (B/Yamagata lineage); (3) 2022-2023*: A/Victoria/2570/2019 (H1N1)pdm09-like virus; B/Michigan/01/2021-like virus (B Victoria lineage); B/Phuket/3073/2013-like virus (B/Yamagata lineage); Serology for H3N2 was not performed as hemagglutinin (HA) antigen of H3N2 was unable to efficiently bind to and agglutinate the red blood cells (16). Hemagglutination Inhibition (HAI) Assay was performed with participant serum, seasonal influenza antigens, and chicken red blood cells (RBC) (Rockland, Catalog # R401-0100) and antibody titer was determined as reported (5, 17, 18). Fold change (FC) response to whole vaccine and each influenza antigen was calculated as the T2/T0 ratio of the Ab titer at T2 and the titer at T0. For each participant, a flu vaccine score was calculated by summing the log_2_-transformed fold change (T2/T0) responses to each of the four influenza antigens that year: Σ (log_2_ (T2/T0)_H1N1_ + log_2_ (T2/T0)_H3N2_ + log_2_ (T2/T0)_B1_ + log_2_ (T2/T0)_B2_) (15, 19). Participants with a vaccine score > 4 were considered vaccine responders (VR) while those with a vaccine score ≤ 4 were considered as vaccine non-responders (VNR). The fold change for the whole vaccine was also calculated as log_2_ (T2/T0) Whole Vaccine.

### Hemagglutinin-specific IgG assay

HA-specific IgG responses to WV and individual antigens A/H1N1 (A/H1), A/H3N2 (A/H3), B/Victoria (B/Vic), and B/Yamagata (B/Yam) were measured using a HA-specific influenza antibody detection by multiplex Luminex assay (HA Luminex) in a subset of participants (n=25 HIV+OP+; n= 22 HIV-OP+; n=57 HIV+OP-; n=43 HIV-OP-). For this assay, serum was diluted 1:200 and incubated overnight at 4 °C with HA-coupled xMAP beads (DiaSorin, Saluggia, Italy) on a shaker. Beads were coupled to HA antigens using a two-step carbodiimide conjugation method, as previously described. After incubation, PE-conjugated anti-human IgG secondary antibodies were added to the serum-bead mixture and incubated for 1 hour on a shaker. Mean fluorescence intensity (MFI) of PE was measured using the FlexMAP 3D instrument (ThermoFisher) and averaged across technical duplicates to provide a relative quantitative measure of antigen-specific HA IgG levels.

### FluoroSpot assay for vaccine-specific antibody-secreting plasmablast

To assess vaccine-induced antigen-specific plasmablast (PB) responses, we measured WV-specific PB at timepoint T1 (day 7 post-vaccination) using a FluoroSpot assay in a subset of study participants (n=36 HIV+OP+; n=50 HIV-OP+; n=47 OP−PWH; n=30 OP−PWoH). Briefly, unstimulated peripheral blood mononuclear cells (PBMCs) were plated at 5 × 10_5_ cells per well in FluoroSpot plates pre-coated with WV antigen, anti-IgM, anti-IgG (positive controls), or culture media alone (negative control). Cells were incubated for 16 hours at 37 °C to allow spontaneous antibody secretion. WV-specific IgM and IgG PB were then determined using isotype-specific detection antibodies and enumerated according to the manufacturer’s instructions. Results are reported as PB per million PBMCs.

### Statistical analysis

Antibody titers were compared between groups using mixed-effects models with Geisser-Greenhouse correction, with matched values at timepoints (T0, T1 and T2), in which timepoints were compared with Tukey’s multiple comparison’s test within each group. Group comparisons of whole vaccine fold change, vaccine score, WV specific IgM and IgG by FluoroSpot and HA-binding IgG were conducted using Kruskal Wallis tests (v9.2.0 GraphPad Prism Inc). Spearman correlations were performed to check the association between WV specific IgM and IgG with vaccine score. Negative binomial regression models for vaccine score and poisson regression models for whole vaccine fold change (negative binomial models for whole vaccine fold change resulted in warnings that suggested poisson regression was a better choice for this data distribution) were constructed (1) to separately examine the relationships between HIV and opioid use on the outcome variables, controlling for sex, race, ethnicity, age, inflammation via cytokine score, baseline influenza titer, flu season and influenza vaccine history, and (2) to investigate other substance use in addition to opioid use. In initial models, we included all two-way interactions among HIV, opioid use, stimulant use, and baseline cytokine score. In addition, interactions between age and baseline cytokine score were included in the initial models. If interactions were not significant, they were removed from the final model. In order to distinguish between associations between HIV and influenza titer versus CD4 and influenza titer, negative binomial regression (vaccine score) and poisson regression (whole vaccine fold change) were performed with CD4 substituting for HIV. CD4 data were available for a subset of the participants; therefore, covariates included in the CD4 models were kept to a minimum in order to maximize sample size for this tailored analysis. Negative binomial regression (MASS), poisson regression (glm) and regression plots (ggeffects) were performed using R and RStudio (v.4.2.1/v.4.2.2; v.2022.07.2). Longitudinal analysis of whole vaccine titers and the sum of log_2_ of the 4 antigen titers at T0 and three subsequent time points (approximately 7, 21-28, and 182 days post-vaccination) was conducted (lme4) using the same covariates. Several forms of the non-linear time relationship were tested and the natural cubic spline with 3 knots showed the best performance (AIC and BIC, lme4).

### Ethics approval

The study was approved by the University of Miami Institutional Review Board (University of Miami IRB # 20200178). Prior to participating in the study, a voluntary signed informed consent for data and sample collection was obtained from each participant.

## Results

### Study population

A total of 243 participants who completed both T0 and T2 visits were included in this study: 39 HIV+OP+, 66 HIV-OP+, 67 HIV+OP-, and 71 HIV-OP-. The median age of participants was 47 years, with a range of 24 to 68 years. Most individuals with OUD, regardless of HIV status, were non-Hispanic White males. In contrast, the majority of individuals without OUD were non-Hispanic Black males (**Table 1**). Among PWH, those with OUD had a similar median baseline CD4 T cell count of 684.5 cells/µL (interquartile range [IQR] 468-927) compared to 672 cells/uL (IQR 486-893) in people without OUD, but values in PWH were significantly lower than in PWoH. The CD4/CD8 ratio followed the same pattern as for CD4 T cells with lower values for PWH than for PWoH. The median baseline Plasma HIV viral load was similar in both the opioid using and non-opioid using groups of PWH, at 20 copies/mL (IQR 20-332) for PWH with OUD and 20 copies/mL (IQR 20-122) for PWH without OUD. The median duration of ART was significantly longer for PWH without OUD, at 188 months (IQR 120-265), compared to 37 months (IQR 24-98) for PWH with OUD.

**Table 1:** Demographic, influenza vaccination history and clinical characteristics of the study participants.

Population	HIV+OP+	HIV-OP+	HIV+OP-	HIV-OP-	Group comparison
*N = 243*	**39**	**66**	**67**	**71**	
Median Age in Years (Range)	49 (29–63)	40 (26-63)	55 (34-68)	46 (24-60)	p < 0.001^a^
Gender					p <0.05^b,†^
Female	15 (38%)	15 (23%)	29 (43%)	29 (41%)	
Male	24 (62%)	51 (77%)	35 (52%)	41 (58%)	
Transgender Woman	0 (0%)	0 (0%)	3 (5%)	1 (1%)	
Race					p <0.0001^b,†^
White or Caucasian	31 (79 %)	49 (74%)	29(43%)	24 (34%)	
Black or African-American	7 (18%)	10 (15%)	35 (52%)	43 (60%)	
Native American	1 (3%)	0 (0%)	0 (0%)	0 (0%)	
Native Hawaiian	0 (0%)	2 (3%)	0 (0%)	0 (0%)	
Other Race	0 (0%)	5 (8%)	3 (5%)	4 (6%)	
Ethnicity					NS^b,†^
Hispanic or Latino	19 (49%)	23 (35%)	24 (36%)	26 (37%)	
Non-Hispanic or Latino	20 (51%)	42 (64%)	43 (64%)	45 (63%)	
Unknown Ethnicity	0 (0%)	1 (1%)	0 (0%)	0 (0%)	
Influenza Vaccine History					p <0.0001^b^
Have Received	31 (81%)	28 (42%)	58 (87%)	47 (66%)	
Have Not Received	6 (14%)	32 (48%)	7 (10%)	18 (25%)	
Unknown Influenza Vaccine History	2 (5%)	6 (10%)	2 (3%)	6 (9%)	
HIV status – median (IQR)					
Baseline CD4 T cell count (cells/µL), *N* = 188	684.5 (468-927)	962 (785-1260)	672 (486-893)	1099 (712-1442)	p < 0.0001^a^
Baseline CD4/CD8 Ratio, *N* = 152	0.70 (0.49-0.79)	1.78 (1.35-2.47)	0.71 (0.44-1.23)	2.16 (1.53-2.91)	p < 0.001^a^
Baseline plasma HIV Viral Loads, RNA copies/mL*	20 (20-332)	NA	20 (20-122)	NA	NS^c^
Duration of ART, months	37 (24-98)	NA	188 (120-265)	NA	p < 0.001^c^

*20, limit of detection; NA, Not Applicable; NS, Not Significant; ^a^Kruskal-Wallis; ^b^Chi-Square; ^c^Mann-Whitney Test; † = Number of participants removed from demographic comparisons due to small cell size (Gender (n=5), Race (n=18), Ethnicity (n=2).

### Influenza-specific antibody responses increase in all four study groups following influenza vaccination

**Figure 2:**
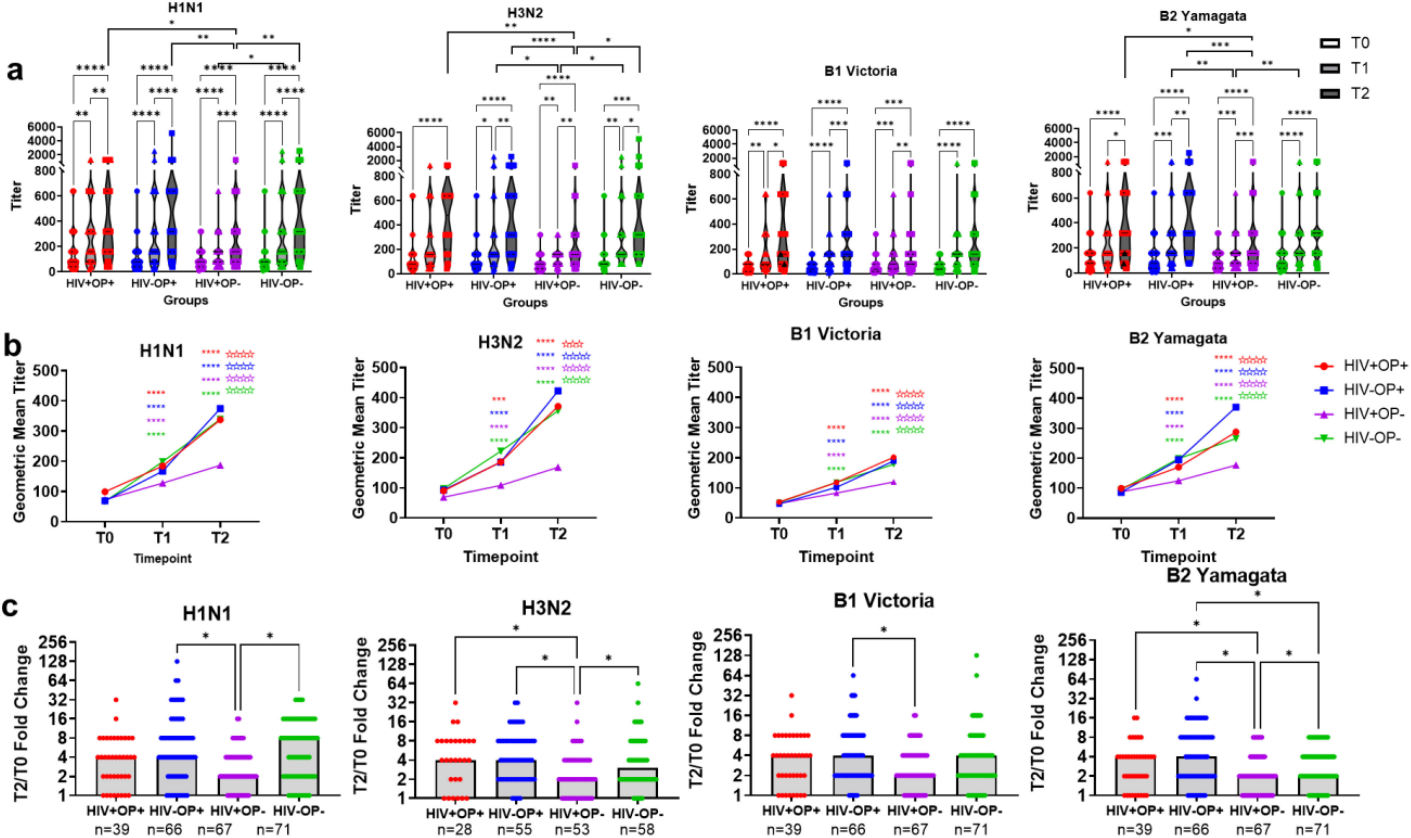
People with OUD, with and without HIV, have positive responses to flu vaccine antigens. **(a)** Truncated violin dot plots with median (solid line) and quartiles (first and third, dashed) for titer expression of influenza antigens (H1N1, H3N2, B1 Victoria, and B2 Yamagata) at T0 (pre-vaccination), T1 (approximately Day 7, post-vaccination) and T2 (approximately Day 21-28, post-vaccination). **(b)** Line graphs displaying geometric mean of antibody antigen titers (H1N1, H3N2, B1 Victoria, and B2 Yamagata) at T0, T1, and T2. Closed stars are comparing T(x) vs T0 and open stars are comparing T2 vs T1 in each group. For **(a)** and **(b)**, a mixed-effect model with Geisser-Greenhouse correction, matched values with time points (T0, T1, T2). Tukey’s multiple comparisons test, with individual variance computed for each comparison. **(c)** Box dot plots with median fold change (T2/T0) expression of influenza antigens H1N1, H3N2, B1 Victoria, and B2 Yamagata. Kruskal-Wallis test with FDR method of Benjamini and Hochberg for multiple comparisons. Red represents HIV+OP+ (H1N1, B1, B2 (n=39); H3N2 (n=28)), Blue represents HIV-OP+ (H1N1, B1, B2 (n=66); H3N2 (n=55)), Purple represents HIV+OP- (H1N1, B1, B2 (n=67); H3N2 (n=53), and Green represents HIV-OP- (H1N1, B1, B2 (n=71); H3N2 (n=58). Adjusted p-values: ****p<0.0001, ***p<0.001, **p<0.01, *p<0.05.

Significant increases in HAI influenza antibody titers to all influenza antigens (**Figure 2a**) and to the WV (**Supplementary Figure S2a**) were observed across all groups from T0 to T2 (p<0.05 – p<0.0001). There were no significant differences between groups for any of the T0 titers (**Figures 2a, b**). When comparing titers between groups, HIV+OP- had significantly lower T1 titers compared to HIV-OP- for H1N1, H3N2, B2 Yamagata, and the WV (p<0.05 – p<0.01) and to HIV-OP+ for H3N2 (p<0.05) and B2 Yamagata (p<0.001). For T2 titers, HIV+OP- had significantly lower responses than HIV+OP+, and HIV-OP+ for H1N1, H3N2, B2 Yamagata and the WV (p<0.05 – p<0.0001; **Figure 2a**; **Supplementary Figure S2a**). No significant differences in B1 Victoria titers were observed between study groups at any timepoint. The geometric mean distribution of titers increased from T0 to T2 in all groups for each antigen and the whole vaccine (**Figure 2b**; **Supplementary Figure S2b**). The geometric means at each time point were similar for HIV+OP+, HIV-OP+, and HIV-OP-, but HIV+OP- consistently had the lowest geometric mean over time. Ab responses, based on T2/T0 fold change (FC), to each influenza seasonal antigens were calculated (**Figure 2c**; **Supplementary Figure S2c**). HIV+OP- had the lowest median fold change compared to the other groups for H3N2, B2 Yamagata and whole vaccine while the difference for B1 Victoria was observed only for HIV-OP+ and the difference for H1N1 was observed only between HIV-OP+ and HIV-OP- groups. Additionally, FC antibody responses to B2 Yamagata and the whole vaccine were lower in HIV-OP- compared to HIV-OP+ (**Figure 2c**; **Supplementary Figure S2c**).

### Opioid use is associated with a higher vaccine response

**Figure 3:**
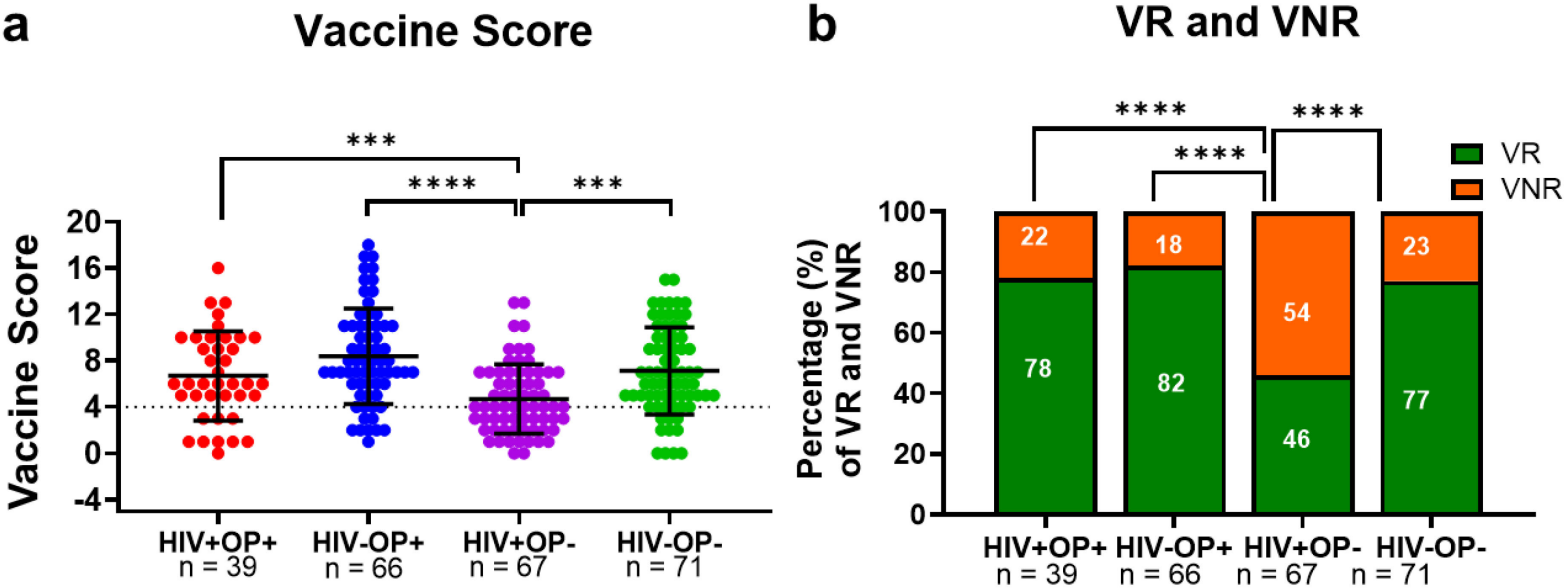
People with OUD, with and without HIV, have similar vaccine scores to people without OUD. **(a)** Scatter dot plots of vaccine score, by group. Individual plots are plotted with median (solid line) and quartiles (first and third, dashed colored lines). Black dotted line distinguished between vaccine responders (>4) and vaccine non-responders (≤4). Non-parametric Kruskal Wallis test was corrected for multiple comparisons by controlling the FDR (original FDR method of Benjamini and Hochberg). Adjusted p-values: ****p<0.0001, ***p<0.001. Red represents HIV+OP+ (n=39, Blue represents HIV-OP+ (n=66), Purple represents HIV+OP- (n=67), and Green represents HIV-OP- (n=71). **(b)** Stacked bar graphs displaying the percentage of vaccine responders (VR) and vaccine non-responders (VNR) per group. Fisher’s exact test for group comparison. Adjusted p-values: ****p<0.0001, ***p<0.001.

The median vaccine score was significantly lower in HIV+OP- (4.0) compared to HIV+OP+ (7.0, *p* < 0.001), HIV-OP+ (8.0, *p* < 0.0001), and HIV-OP- (6.0, *p* < 0.001) (**Figure 3a**). The distribution of vaccine responders (VR) and non-responders (VNR) also differed significantly across the four groups (*p* < 0.0001), with HIV+OP- exhibiting the lowest percentage of VR (46%) and highest percentage of VNR (54%) compared to HIV+OP+ (78% VR, 22% VNR, *p* < 0.001), HIV-OP+ (82% VR, 18% VNR, *p* < 0.0001), and HIV-OP- (77% VR, 23% VNR, *p* < 0.001) (**Figure 3b**).

In regression analysis, T0 titers were negatively associated with both vaccine score (β = -0.08, *p* < 0.001) and log_2_-transformed whole vaccine fold change (β = -0.28, *p* < 0.001) (**Table 2**). Flu season had no significant association with either vaccine score or log_2_-transformed whole vaccine fold change. As expected, recency of previous influenza vaccination was inversely associated with vaccine score. Those who self-reported as never having been vaccinated had a greater influenza vaccine response compared to those who had been vaccinated within the previous two years (β=0.26, *p* < 0.05) and, likewise, those who had been vaccinated more than two years ago had a greater response compared to those who had been vaccinated within the previous two years (β=0.38, *p* < 0.001) (**Table 2**). The overall Type III test for recency of influenza vaccination was not significant (*p >*0.05) for log_2_-transformed WV vaccine fold change, but the coefficient for having been vaccinated more than two years ago was significant (β = 0.36, *p* < 0.05) (**Table 2****).** Other demographic characteristics such as sex, race, and ethnicity did not show an association with antibody responses following vaccination in our cohort (**Table 2**).

**Table 2:** Regression predictors of vaccine score and whole vaccine fold change (T2/T0) response.

Variable	Vaccine Score Coefficient (SE)^a^ n=181	Whole Vaccine Fold Change (T2/T0) Coefficient (SE)^a^ n=181
Intercept	4.18 (0.39) ***	2.34 (0.63) ***
Baseline Titer	-0.08 (0.01) *** ^c^	-0.28 (0.07) *** ^b^
Flu Season (season 2)	0.11 (0.08)	0.12 (0.14)
Never previously received influenza vaccine (ref=received influenza vaccine < 2 years previously)	0.26 (0.10)*	0.26 (0.19)
Received influenza vaccine > than 2 years previously (ref=received influenza vaccine < 2 years previously)	0.38 (0.10)***	0.36 (0.17)*
Sex at birth (Male)	-0.11 (0.07)	-0.14 (0.13)
Ethnicity (Hispanic)	-0.09 (0.08)	-0.05 (0.15)
Race (African American) (ref=White)	-0.06 (0.09)	-0.11 (0.16)
Race (Other) (ref=White)	-0.21 (0.18)	-0.17 (0.32)
Age	-0.01 (0.00)	-0.00 (0.01)
HIV status (HIV+)	-0.45 (0.10) ***	-0.55 (0.19) **
Opioid Use (OP+)	-0.21 (0.14)	-0.04 (0.20)
Stimulant Use (ST+)	-0.07 (0.11)	0.15 (0.14)
Marijuana Use (THC+)	0.03 (0.08)	0.09 (0.14)
Alcohol Use (ETG+)	-0.14 (0.09)	-0.23 (0.17)
Benzodiazepine Use (BZO+)	-0.01 (0.11)	0.01 (0.20)
Smoking (smoke+)	-0.07 (0.08)	-0.06 (0.16)
Baseline Cytokine Score	-0.71 (0.25)**	0.10 (0.13)
Interaction: HIV status and Opioid Use	0.37 (0.14) **	0.55 (0.26) *
Interaction: HIV status and Stimulant Use	0.36 (0.16)*	
Interaction: Age and Baseline Cytokine Score	0.02 (0.01)**	

a p-values: ***p<0.001, **p<0.01, *p<0.05.

b Baseline Titer for Whole Vaccine.

c Baseline Titer Score for All Antigens.

### Interactions among HIV, opioid use, and other variables

**Figure 4:**
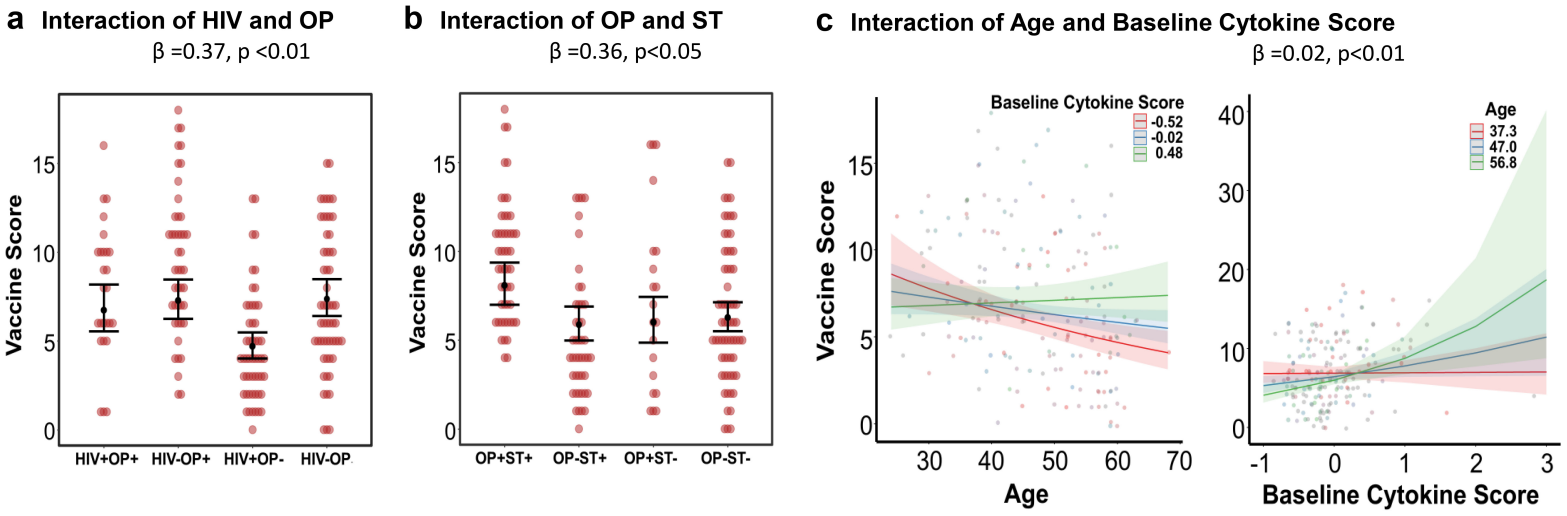
Significant interaction relationships found with vaccine score as modeled in a negative binomial regression (see text and **Table 2** for details). Shown are interaction effects while controlling for other covariates in the regression model. Individual plots show predicted mean and 95% confidence intervals or bands. Individual data points are the raw values. **(a)** HIV by OP interaction; **(b)** OP by Stimulant (ST) interaction; **(c)** age by baseline cytokine score interaction. Two plots are shown for the same age by baseline cytokine interaction effect to display continuous age by three levels of baseline cytokine score and continuous baseline cytokine score for 3 ages.

There was a significant interaction between HIV status and opioid use associated with vaccine score (β = 0.37, p<0.01, **Table 2**). After controlling for all variables, HIV+OP- had the lowest vaccine score compared to HIV+OP+, HIV+OP- and HIV-OP+ (**Figure 4a**). A significant interaction between HIV and opioid use was also observed for log_2_-transformed whole vaccine fold change (β = 0.55, p<0.05, **Table 2**). Similar to vaccine score, HIV+OP- had the lowest whole vaccine fold change response compared to HIV+OP+, HIV-OP+ and HIV-OP- (**Supplementary Figure S3a**). Additionally, interactions between opioid use and stimulant use was found (β = 0.36, p<0.05, **Table 2**) whereby those using both opioids and stimulants had the highest vaccine score (**Figure 4b**). Interaction between age and baseline cytokine score was significant for vaccine score (β = 0.02, p<0.01, **Table 2****).** The relationship between baseline cytokine score, age, and vaccine score indicated that the highest vaccine scores were found in older individuals with higher cytokine scores or young individuals with lower cytokine scores (**Figure 4c**). Other significant associations with vaccine score and log_2_-transformed whole vaccine fold change (**Table 2**) are illustrated in **Supplementary Figure S3**. Absolute CD4 counts were significantly lower among PWH, regardless of opioid use status (**Table 1****).** In regression models, CD4 count was significantly associated with vaccine score (β = 0.0003, *p* < 0.01), but not with log_2_-transformed WV fold change (β = 0.00007, *p* > 0.05 (**Supplementary Table S2**).

### Hemagglutinin-specific IgG

Significant increases in HA-specific IgG responses to A/H1, A/H3, B/Victoria (B/Vic), and B/Yamagata (B/Yam) were observed from day 0 to day 28 post-vaccination (dpv) across all four groups (**Supplementary Figure S4**). Between day 28 and day 180, IgG levels declined significantly for all antigens in every group. Notably, A/H1-specific IgG remained elevated above baseline at day 180 across all groups, independent of HIV status or opioid use, whereas A/H3-specific IgG returned to baseline exclusively in the HIV+OP+ group. At day 180, IgG levels against all antigens were significantly lower in the HIV+OP- group compared to all other groups, indicating an HIV-associated impairment in antibody persistence. Similarly, at the T2 time point, IgG levels were significantly lower in HIV+OP- compared to HIV-OP- across all antigens.

### Vaccine-specific antibody-secreting plasmablast response

We next analyzed the vaccine-induced IgM and IgG plasmablast responses at day 7 post-vaccination using a FLUOROspot assay (**Supplementary Figure S5****).** WV-specific IgM-PB were significantly lower in HIV+OP- compared to all other groups. Additionally, IgM PB were higher in HIV-OP+ compared to HIV+OP-. The WV-specific IgG-PB response was significantly lower in HIV+OP- compared to HIV-OP+, with a trend toward higher responses in the other groups relative to HIV-OP-. When all four groups were analyzed together, WV-specific IgM and IgG PB responses correlated with the composite vaccine score (IgM: r = 0.189, p = 0.26; IgG: r = 0.224, p = 0.008). This correlation was primarily driven by PB in the HIV+ groups (IgM: r = 0.461, p < 0.0001; IgG: r = 0.307, p = 0.008), and was not observed in the opioid groups. These findings are consistent with the lower HAI antibody responses observed in the HIV+OP- group.

### Longitudinal analysis of Ab response over time

**Figure 5:**
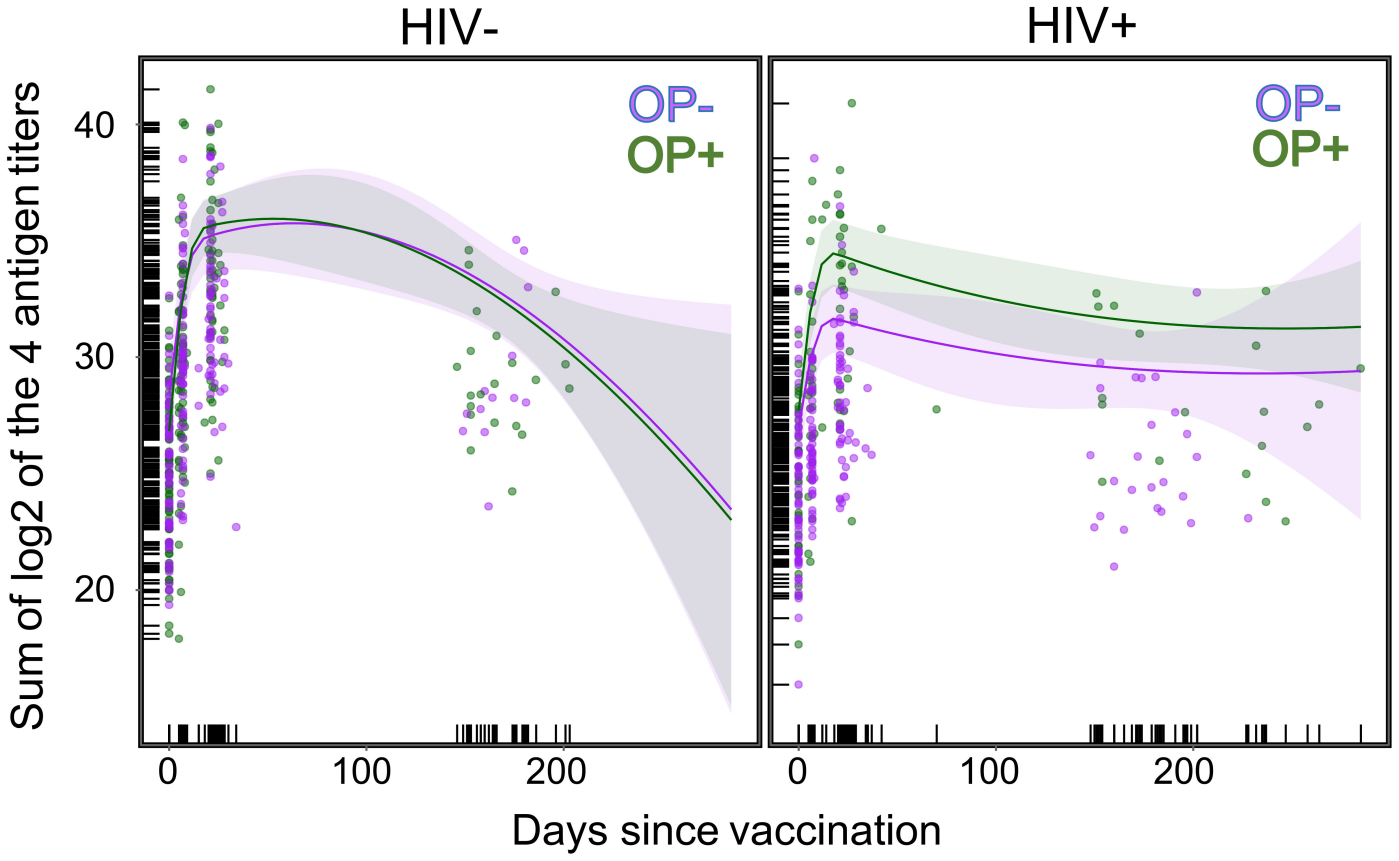
Scatter plots compare antigen titers over days since vaccination for HIV- and HIV+ individuals. Results of longitudinal model of the sum of log_2_ of the 4 antigen titers in relation to HIV status and Opioid use. 95% confidence bands shown for OP+ (green) and OP- (purple). Curves shown are predicted values from the Natural Cubic Spline regression model and individual data points are the raw values (details in text, **Table 3**).

Similar to vaccine score and fold change findings, there was a significant interaction between HIV and opioid use in longitudinal analyses, with HIV+OP+ participants showing Ab response trajectories similar to PWoH individuals (**Table 3**, **Supplementary Table S3**, **Figure 5**, **Supplementary Figure S6**). HIV+OP- individuals had the lowest vaccine response over time (**Figure 5**, **Supplementary Figure S6**). Tests of differences among modeled means at the planned 7-, 28-, and 182-day titers indicate that, among participants with HIV, opioid use was associated with a significantly higher sum of the log_2_ of the four antigen titers at day 7, day 28, and day 182 (t ratio=-3.24, df=282, p<0.002; t ratio=-3.82, df=338, p<0.0002; t ratio=-2.32, df=583, p<0.03, respectively). Similarly, planned 7-, 28-, and 182-day comparisons indicate that, among participants with HIV, opioid use was associated with a significantly higher whole vaccine titer at day 7, day 28, and day 182 (t ratio=-3.44, df=351, p<0.0007; t ratio=-3.74, df=436, p<0.0002; t ratio=-2.21, df=663, p<0.03, respectively). Among PWoH, there were no differences at day 7, day 28, or day 182 among people who used opioids and those that did not use opioids (t ratio=0.23, df=293, p=0.82; t ratio=-0.57, df=336, p=0.57; t ratio=0.36, df=648, p=0.72, respectively, for sum of log_2_ of the 4 antigen titers; t ratio=0.21, df=381, p=0.84; t ratio=-0.34, df=439, p=0.73; t ratio=0.74, df=761, p=0.46, respectively, for log_2_-transformed whole vaccine titer). Unlike the vaccine score and log_2_-transformed whole vaccine fold change analyses, we did find significant associations with age, ethnicity, and race with the summed titers of the individual antigens but not with log_2_-transformed whole vaccine titer (**Table 3**, **Supplementary Table S3**).

**Table 3:** Longitudinal analysis of Vaccine Score at baseline and 3 timepoints after baseline (approximately 7, 21-28, and 182 days post-vaccination).

Fixed effects^a^:	Estimate	Std. Error	df	t value	p value^b^
(Intercept)	27.41	1.24	233.52	22.02	0.00***
HIV Status (HIV+)	-1.47	0.58	326.55	-2.53	0.01*
Age	-0.05	0.02	223.27	-2.22	0.03*
Ethnicity (Hispanic)	-1.30	0.47	222.13	-2.77	0.01**
Race (Black/African American)	-0.25	0.49	218.07	-0.50	0.62
Race (Other)	1.77	0.79	230.61	2.23	0.03*
Sex at birth (Male)	-0.53	0.38	220.37	-1.37	0.17
Never previously received influenza vaccine (ref=received influenza vaccine < 2 years previously)	-0.31	0.53	218.32	-0.58	0.56
Received influenza vaccine > than 2 years previously (ref=received influenza vaccine < 2 years previously)	-0.01	0.48	218.70	-0.01	0.99
Flu Season (2)	1.26	0.38	223.67	3.30	0.00**
Opioid use (OP+)	-1.59	0.64	298.40	-2.48	0.01*
Stimulant use (ST+)	0.72	0.41	215.15	1.74	0.08
Benzodiazepine use (BZO+)	0.03	0.58	223.65	0.06	0.95
Marijuana use (THC+)	0.54	0.41	216.50	1.32	0.19
Alcohol use (ETG+)	-0.67	0.47	216.87	-1.42	0.16
ns(days, 3) time 1	9.69	5.27	503.95	1.84	0.07
ns(days, 3) time 2	-10.79	10.29	504.46	-1.05	0.29
ns(days, 3) time 3	-46.55	24.49	504.27	-1.90	0.06
Interaction: HIV Status : Opioid use	2.35	0.76	224.49	3.11	0.00**
HIV status: ns(days, 3) time 1	-11.48	5.18	510.99	-2.22	0.03*
HIV status: ns(days, 3) time 2	17.86	9.71	511.21	1.84	0.07
HIV status: ns(days, 3) time 3	50.07	23.20	511.09	2.16	0.03*
OP+:ns(days, 3) time 1	-0.18	4.56	510.00	-0.04	0.97
OP+:ns(days, 3)time 2	2.88	8.01	511.80	0.36	0.72
OP+:ns(days, 3) time 3	0.67	19.31	511.59	0.04	0.97

a Random effects: Participant, variance=5.10, SD = 2.26; Residual, variance=5.36, SD = 2.32.

b p value: ***p<0.001, **p<0.01, *p<0.05.

## Discussion

This study investigated the influenza vaccine-induced antibody response in PWH to understand how chronic opioid use impacts their ability to respond as compared to PWoH with and without OUD. Influenza vaccines are now universally recommended in the US and are considered particularly important for individuals based on age, lifestyle, occupation, and immune status (20–22). In PWH, vaccination is highly recommended to lower the risk of infectious morbidity (23, 24). Previously, we and others have shown that despite virologic control with ART, PWH exhibit diminished antibody responses to influenza vaccines (5, 6, 17, 25, 26), but there is a gap in understanding the effect of OUD in PWH. Our cohort is based in Miami, a city with one of the highest annual rates of newly diagnosed HIV, with 39.3 cases per 100,000 people (27), and a high incidence of 11.3 cases per 100,000 people for opioid related deaths (28). The PWH in the OPIS study were virally suppressed with ART at study entry (4) and the IDEA clinic provided health services for people with OUD, such as syringe needle exchange to reduce transmission of infectious disease (29). Contrary to expectations, this study showed that chronic opioid use did not adversely affect the antibody response to influenza vaccination within this study cohort; instead, opioid use in PWH augmented the antibody response in comparison to non-opioid-using PWH, confirming previous preliminary findings (15). We found that the HIV+OP+ group exhibited a significantly higher fold change response than HIV+OP- participants for H3N2, B1 Victoria, B2 Yamagata, and whole vaccine. We reached this conclusion after adjusting for demographics, baseline titers, previous vaccination history, and concomitant use of other substances and noted significant interactions between HIV and opioids. Overall, our study provides new insights into the effects of opioid use on humoral responses to influenza vaccination in PWH, which have not been previously reported. The underlying mechanisms by which opioid use enhanced influenza vaccine induced antibody responses in PWH warrant further investigation.

Immune dysfunction in virally suppressed PWH is marked by a lower antibody response to influenza vaccination, along with alterations in immune cell dynamics that hinder vaccine effectiveness in this group (6, 17, 30, 31). Consistent with our earlier studies, HIV+OP- participants showed the lowest T0 to T2 geometric mean titer response, and T2/T0 fold change response to all four antigens (H1N1, H3N2, B1 Victoria and B2 Yamagata) (5, 17, 32). We have further refined the criteria for defining people as vaccine responders and non-responders by utilizing a vaccine score (15, 19). Based on the vaccine score (15, 19), PWH without OUD had the lowest percentage of vaccine responders and the highest percentage of vaccine non-responders, which aligns with our previous observations (5). Several studies have investigated the underlying immune defects in PWH and have revealed both qualitative and quantitative impairments in pTfh cells, B cells, and antigen-presenting cells (APCs), as well as in their interactions essential for effective antibody production (6, 17, 18, 25, 26, 33). These defects include increased frequencies of circulating inflammatory monocytes, reduced expansion of antigen-responsive pTfh cells with diminished IL-21 induction and elevated IL-2 expression, and evidence of premature immune senescence within the B cell compartment (6, 17, 18, 25, 26, 33). The key question, therefore, is how opioid exposure modulates or potentially counteracts these immune impairments.

There is conflicting data about the immunologic effects of opioids (34–36). Exogenous and endogenous opioids exert immunosuppressive effects on both innate and adaptive immunity, altering cytokine production, immune cell function, and susceptibility to infections (37). Single-cell RNA sequencing (scRNA-seq) of peripheral blood mononuclear cells from opioid-dependent individuals and controls revealed that chronic opioid use broadly suppresses antiviral gene programs in naïve monocytes and other immune cell types (38). Moreover, studies have shown that fentanyl increases replication of HIV and proviral burden in several cell types (39, 40). Opioids can either directly affect each of the immune cells or mediate downstream effects leading to immunosuppression. For instance, opioids can inhibit the TCR signaling cascade of T cells through the PKA-Cbp/PAG/Csk-Lck pathway, resulting in reduced T cell activation and IL-2 induction (41), thereby decreasing T-B cell interaction and lowering antibody secretion. In animal models, a Mu-opioid receptor agonist induced IgM and IgG antibody production in the peripheral blood (42). The impact of chronic opioid use on influenza vaccine-induced antibody responses has been previously examined only in a small study with a limited number of young PWoH, who used heroin (43) and showed a trend towards enhanced HAI Ab titer compared to those who did not use heroin, but did not show statistically significant differences (43).

The day 7 plasmablast surge reflects a well-characterized, transient increase in circulating antibody-secreting cells that peaks approximately one week after influenza vaccination. These plasmablasts, derived from activated memory B cells, serve as an early correlate of the humoral immune response and are predictive of subsequent antibody titers. Their frequency and phenotype provide important insights into the magnitude and quality of vaccine-induced B cell activation (18, 44–46). The HIV+OP- group exhibited a significantly lower IgG and IGM plasmablast response at day 7. Consistent with previous reports (17, 18), we found comparable levels of WV-specific IgM and IgG plasmablasts at day 7 post-vaccination between OP+ and OP- PWoH. Notably, the plasmablast response correlated with the vaccine score in PWH but not in opioid users, suggesting that possible defects in the plasmablast response may contribute to the lower antibody responses in PWH. In HIV+OP+ participants, opioid use may mitigate this defect and enhance the plasmablast response.

We also examined HA-specific IgG responses to the influenza vaccine, which reflect the binding antibody response to the HA antigen and provide insight into the overall breadth and magnitude of the vaccine-induced B-cell response. Consistent with the HAI titers, analysis of HA-binding IgG responses to H1N1, H3N2, B1 Victoria, and B2 Yamagata revealed a significant post-vaccination increase, further reinforcing the observed HAI responses. We conducted analyses to understand factors underlying enhancement in vaccine-induced antibody responses among opioid users. Several factors can influence vaccine responses, such as demographics, the timing of the flu season, recency of previous vaccination, baseline titers, substance use, inflammation, and HIV infection. Additionally, age, sex, race, socioeconomic status, and comorbidities add variability that can either obscure or enhance vaccine responsiveness across different populations (47–50). In regression modeling, we statistically controlled for key variables and assessed interactions. Demographic characteristics such as sex, race, and ethnicity did not show a consistent association with antibody responses following vaccination in our cohort. As expected, recent previous vaccination (47), and higher baseline HAI titers were negatively associated with vaccine responses, while absolute CD4 counts were positively associated. Additionally, we did not observe any consistent effect of a specific flu season on vaccine score or log_2_-transformed whole vaccine FC, indicating that inter-seasonal variation had minimal impact on vaccine responsiveness. In contrast, the recency of prior influenza vaccination showed a clear relationship with immune response magnitude. Participants who had not received an influenza vaccine in the past two years or were never vaccinated exhibited significantly higher vaccine responses compared to those vaccinated within the previous two years. The positive association for individuals vaccinated more than two years prior suggests partial recovery of responsiveness with longer intervals between vaccinations. Together, these findings support the concept that recent annual vaccination may attenuate antibody responses, whereas longer intervals between vaccinations are associated with more robust immune boosting. The results presented here confirm previous observations that repeated influenza vaccination over multiple years lowers overall immunogenicity (51, 52).

HIV negatively impacts the influenza titer response; however, when PWH chronically use opioids, the interaction between chronic HIV infection-associated immune status and opioid use appears to enhance antibody responses, suggesting a beneficial effect of opioids in increasing Ab response in PWH. However, this effect was not observed in PWoH, where antibody titers were similar for both people who use opioids and those who did not. This interaction was so evident that the peak antibody titer at day 28 post-vaccination was comparable between HIV+OP+ and the PWoH groups. We also reported that opioid use exacerbated inflammation in PWH ^4,16^. The relationship between inflammation (cytokine score) and vaccine responsiveness showed two distinct age-dependent patterns: older individuals with higher cytokine scores and younger individuals with lower cytokine scores exhibited stronger antibody responses. This finding suggests that moderate baseline inflammation may compensate for immune dysfunction in older adults, whereas elevated inflammation in younger individuals may be detrimental, reflecting dysregulation that blunts vaccine responsiveness.

Longitudinal analysis of titers over time demonstrated a significant interaction between HIV status and opioid use in shaping vaccine responses over time. Notably, HIV+OP+ exhibited antibody response trajectories similar to those of PWoH, suggesting that opioid use in the context of HIV may be associated with a more preserved or enhanced humoral response. In contrast, HIV+OP- showed the lowest vaccine responses across all time points, indicating that opioid use may differentially impact immune function in PWH. Among PWH, opioid use was consistently linked to significantly higher antibody titers at days 7, 28, and 182 post-vaccination, and these effects were statistically robust and sustained over the 6-month follow-up period. Conversely, in PWoH, opioid use did not significantly alter vaccine-induced antibody responses at any time point compared to HIV-OP- group, indicating that the effect of opioids may be specific to improve the immunologic context of HIV infection. Further studies are warranted to explore how opioids interact with HIV-related immune alterations to influence B and T cell helper function to sustain vaccine-induced immunity.

Possible mechanisms underlying the opioid-mediated enhancement of influenza vaccine responses in PWH likely involve complex immunomodulatory effects of opioids. Although differences in immune reconstitution do not explain the observed effects, as absolute CD4^+^, CD8^+^, and CD4/CD8 ratios were comparable between HIV+OP+ and HIV+OP- individuals. We have shown that chronic opioid use upregulates members of the TNF superfamily (TNFRSF) molecules in plasma (15). HIV+OP+ participants displayed significantly higher plasma levels of co-stimulatory molecules (OX40, OX40L, CD40, CD40L, 4-1BB, 4-1BBL) compared to HIV+OP- participants (15), indicating heightened baseline activation of TNFRSF co-stimulatory pathways that may create an immune-primed state and enhance vaccine responsiveness. This elevated co-stimulation reflects increased engagement of pathways essential for T-cell activation, Tfh differentiation, germinal center function, and B-cell class switching. Upregulated OX40/OX40L, CD40/CD40L, and 4-1BB/4-1BBL signaling may therefore act as a compensatory mechanism that strengthens T–B interactions and improves IgG production. Thus, chronic immune activation that is typically detrimental in HIV may, in this context, confer functional benefits by promoting a pre-activated immune milieu that enhances vaccine-induced antibody responses. Further studies are needed to understand whether opioids directly modulate the functional state of Tfh cells or B cells through opioid receptor mediated signaling, potentially altering cytokine production or co-stimulatory molecule expression that influences B cell activation, class switching, and plasma cell differentiation. This modulation may transiently create a more permissive immune environment that offsets HIV-related defects in Tfh–B cell interactions, suggesting a paradoxical immunostimulatory effect of chronic opioid exposure on vaccine responsiveness.

One of the key strengths of this study is the use of a unique cohort that enables the examination of the combined effects of opioid use and HIV status on influenza vaccine responses across multiple time points. Through rigorous statistical analyses, including multivariable regression modeling, we identified novel evidence suggesting enhanced immune responses associated with opioid use in PWH, highlighting a potential interaction between opioid exposure and HIV infection. However, our study was limited by the relatively small sample size within the HIV+OP+ group, which may reduce statistical power and limit the detection of subtle yet biologically meaningful effects. In addition, detailed information on opioid dosage and duration of use was not available, which restricts the generalizability of our findings across the duration of opioid exposures. Potential confounders such as nutritional status, comorbidities, and the use of immunosuppressive medications were not fully controlled for and could have influenced immune outcomes.

In conclusion, this study demonstrates that chronic opioid use does not impair but rather enhances influenza vaccine-induced antibody responses in PWH. Contrary to our initial hypothesis that OUD would worsen HIV-associated immune dysfunction resulting in impaired vaccine responses, we found that PWH with OUD exhibited significantly higher fold-change in antibody responses compared to PWH without OUD. These effects remained significant after adjusting for demographic factors, previous vaccination history, baseline titers, and concomitant substance use, with regression modeling confirming a strong interaction between HIV and opioid use on humoral immune outcomes. Although OUD has been linked to increased systemic inflammation, often presumed to compromise vaccine efficacy, our findings suggest a more complex immunologic relationship in which opioid exposure may partially mitigate the humoral immune defects typically associated with HIV infection. This unexpected enhancement of influenza vaccine responsiveness among PWH using opioids underscores the need for mechanistic studies to define how chronic opioid exposure influences immune signaling and B cell function in the context of HIV. Overall, these results provide new insights into the interplay between opioid use and HIV infection and highlight the importance of understanding how chronic substance use modulates vaccine-induced immunity in vulnerable populations.

## Data Availability

The raw data supporting the conclusions of this article will be made available by the authors, without undue reservation.
