# Supplementary material for: Chronic opioid use is associated with higher antibody response to influenza vaccination in people living with HIV
Source: Front Immunol. 2025 Dec 17;16:1686103. doi: 10.3389/fimmu.2025.1686103 (PMC12753940; doi:10.3389/fimmu.2025.1686103)
Supplement: Supplementary file 1 [file DataSheet1.pdf]

## Supplementary Information

### Supplementary Tables:

**Table S1:** Urine Drug Screen information:

| Population | HIV+OP+ | HIV-OP+ | HIV+OP- | HIV-OP- |
|---|---|---|---|---|
| *N* | **39** | **66** | **67** | **71** |
| # of Substances – Median (IQR) | 2 (2-3) | 3 (2-3) | 1 (0-2) | 1 (0-2) |
| **Opioid** | | | | |
| Fentanyl | 44 % | 80 % | 0 % | 0 % |
| Buprenorphine | 46 % | 20 % | 0 % | 0 % |
| Morphine* | 15 % | 20 % | 0% | 0 % |
| Methadone | 5 % | 6 % | 0 % | 0 % |
| Oxycodone | 5 % | 3 % | 0 % | 0 % |
| Tramadol | 0% | 2 % | 0 % | 0 % |
| **Stimulant** | | | | |
| Cocaine | 54 % | 65 % | 40 % | 31 % |
| Amphetamine | 10 % | 6 % | 6 % | 1 % |
| Methamphetamine | 5 % | 9 % | 4 % | 3 % |
| MDMA** | 0 % | 9 % | 1 % | 3 % |
| **Other Substances** | | | | |
| Benzodiazepines | 21 % | 15 % | 3 % | 10 % |
| Cannabinoid | 21 % | 32 % | 27 % | 44 % |
| Ethyl-Glucuronide | 15 % | 15 % | 19 % | 34 % |
| Barbiturates | 0 % | 0 % | 0 % | 0 % |

*Also a metabolite of Heroin, **MDMA = Ecstasy

**Table S2**. Regression predictors of vaccine score and whole vaccine fold change (T2/T0) response in models including CD4.

| Variable | Vaccine Score Coefficient (SE)[a] n=141 | Whole Vaccine Fold Change (T2/T0) Coefficient (SE)[a] n=188 |
|---|---|---|
| Intercept | 2.97 (0.34)*** | 1.63 (0.51)** |
| Baseline Titer | -0.08 (0.01)*** | -0.29 (0.07)*** |
| Flu Season (2) | 0.31 (0.11)** | 0.19 (0.18) |
| Flu Season (3) | | 0.13 (0.23) |
| Flu vaccine history (never) | 0.35 (0.12)** | 0.37 (0.17)* |
| Flu vaccine history (> 2 years) | 0.39 (0.11)*** | 0.34 (0.17)* |
| Opioid Use | 0.27 (0.09)** | 0.18 (0.13) |
| CD4 | 0.0003 (0.00009)** | 0.00007 (0.00008) |

[a]p-values: ***p<0.001, **p<0.01, *p<0.05

**Table S3:** Longitudinal analysis of whole vaccine titer at baseline and 3 timepoints after baseline (approximately 7, 21-28, and 182 days post-vaccination).

| Fixed effects[a]: | | | | | |
|---|---|---|---|---|---|
| | **Estimate** | **Std. Error** | **df** | **t value** | **p value[b]** |
| (Intercept) | 7.02 | 0.31 | 303.89 | 22.36 | 0.00*** |
| HIV Status (HIV+) | -0.19 | 0.14 | 395.96 | -1.33 | 0.19 |
| Age | -0.01 | 0.01 | 295.46 | -1.81 | 0.07 |
| Ethnicity (Hispanic) | -0.14 | 0.12 | 295.70 | -1.18 | 0.24 |
| Race (Black/African American) | -0.05 | 0.13 | 283.25 | -0.41 | 0.69 |
| Race (Other) | 0.32 | 0.20 | 302.73 | 1.60 | 0.11 |
| Sex at birth (Male) | -0.17 | 0.10 | 285.31 | -1.79 | 0.07 |
| Flu vaccine history (never) | 0.16 | 0.13 | 279.22 | 1.23 | 0.22 |
| Flu vaccine history (> 2 years) | 0.07 | 0.12 | 279.60 | 0.61 | 0.54 |
| Flu Season (2) | -0.03 | 0.11 | 289.04 | -0.27 | 0.79 |
| Flu Season (3) | 0.38 | 0.13 | 291.73 | 2.89 | 0.00** |
| Opioid use (OP+) | -0.33 | 0.16 | 374.70 | -2.09 | 0.04* |
| Stimulant use (ST+) | 0.05 | 0.10 | 281.08 | 0.50 | 0.62 |
| Benzodiazepine use (BZO+) | -0.09 | 0.14 | 292.91 | -0.65 | 0.52 |
| Marijuana use (THC+) | 0.07 | 0.10 | 282.49 | 0.66 | 0.51 |
| Alcohol use (ETG+) | -0.09 | 0.12 | 284.16 | -0.76 | 0.45 |
| ns(days, 3) time 1 | 1.78 | 0.65 | 600.05 | 2.73 | 0.00** |
| ns(days, 3) time 2 | 1.40 | 0.27 | 599.99 | 5.10 | 0.00*** |
| ns(days, 3) time 3 | -2.49 | 0.84 | 602.00 | -2.98 | 0.00** |
| Interaction: HIV status:Opioid use | 0.61 | 0.19 | 296.31 | 3.22 | 0.00** |
| HIV status: ns(days, 3) time 1 | -1.12 | 0.70 | 611.44 | -1.60 | 0.11 |
| HIV status: ns(days, 3) time 2 | -0.42 | 0.29 | 604.24 | -1.44 | 0.15 |
| HIV status: ns(days, 3) time 3 | 1.20 | 0.87 | 612.28 | 1.38 | 0.17 |
| OP+:ns(days, 3) time 1 | -0.04 | 0.63 | 601.73 | -0.06 | 0.96 |
| OP+:ns(days, 3)time 2 | 0.53 | 0.25 | 596.16 | 2.07 | 0.04* |
| OP+:ns(days, 3) time 3 | -0.01 | 0.73 | 602.46 | -0.02 | 0.99 |

[a] Random effects: Participant, variance=0.43, SD=0.66; Residual, variance=0.36, SD=0.60

[b] p value: ***p<0.001, **p<0.01, *p<0.05

## Supplementary Figures:

### Figure S1

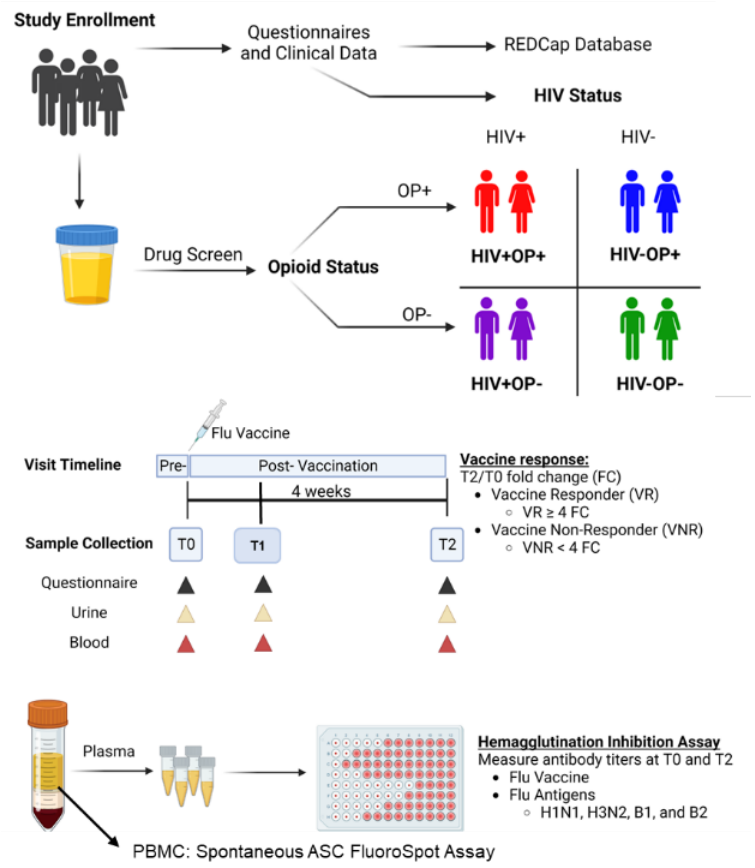


**Figure S1**: Study schema, vaccination and sample collection

**Figure S2**

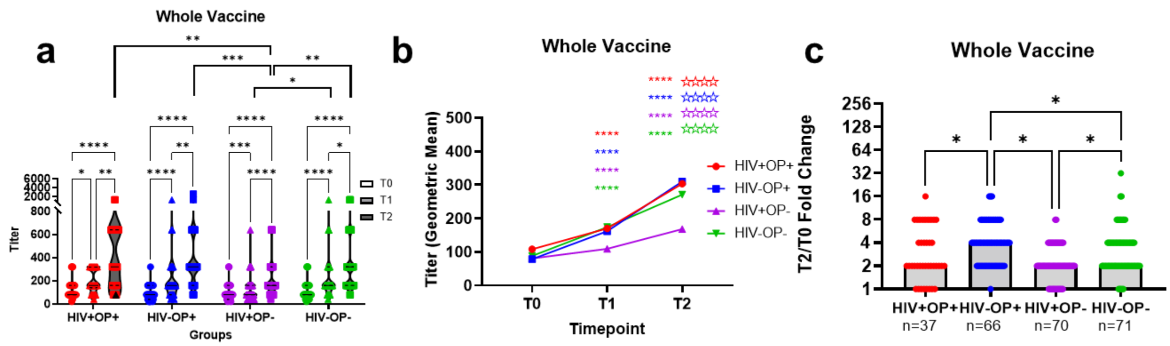


**Figure S2. People with OUD, with and without HIV, have positive responses to whole vaccine:** (a) Truncated violin dot plots with median (solid line) and quartiles (first and third, dashed) for titer expression of whole vaccine at T0 (pre-vaccination), T1 (Day 7, post-vaccination) and T2 (Day 21-28, post-vaccination). Red represents HIV+OP+ n=37), Blue represents HIV-OP+ n=55), Purple represents HIV+OP- (n=70); and Green represents HIV-OP- (n=71). Mixed-effect models with Geisser-Greenhouse correction, matched values with time points (T0, T1, T2). Tukey's multiple comparisons test, with individual variance computed for each comparison. Adjusted p-values: ****p<0.0001, ***p<0.001, **p<0.01, *p<0.05. (b) Line graphs displaying geometric mean titer for whole vaccine at T0, T1, and T2. Red represents HIV+OP+ n=39), Blue represents HIV-OP+ n=55), Purple represents HIV+OP- (n=70); and Green represents HIV-OP- (n=71). Mixed-effect models with Geisser-Greenhouse correction, matched values with time points (T0, T1, T2). Tukey's multiple comparisons test, with individual variance computed for each comparison. Adjusted p-values: ****p<0.0001, ***p<0.001, **p<0.01, *p<0.05. Closed stars are comparing T(x) vs T0 and open stars are comparing T2 vs T1 in each group. (c) Box dot plots with median fold change (T2/T0) response to whole vaccine. Kruskal-Wallis test with FDR method of Benjamini and Hochberg for multiple comparisons. ***p<0.001, **p<0.01, *p<0.05. d)

## Figure S3

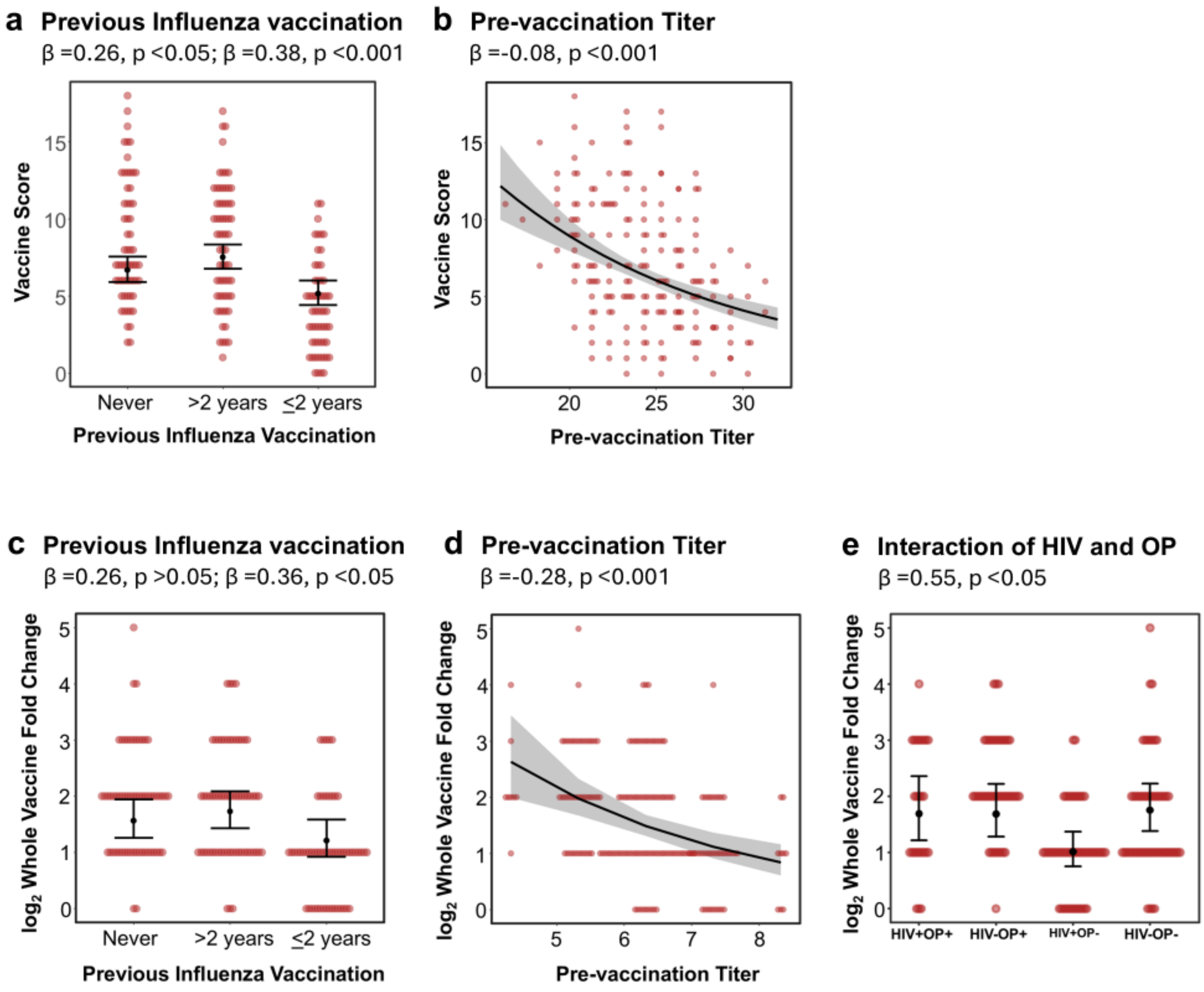


**Figure S3. Significant relationships were found with vaccine score and log-2 transformed whole vaccine fold change.** Shown are variable effects while controlling for other covariates in the regression models. Individual plots show the predicted mean and 95% confidence intervals or bands. Individual data points are the raw values. (a-b) Significant relationships found with vaccine score as modeled in a negative binomial regression (see text and Table 2 for details) for a), previous flu vaccination, and b), pre-vaccination titer. (c-d) Significant relationships found with log2 whole vaccine fold change as modeled in a Poisson regression (see text and Table 2 for details) c), previous flu vaccination, and d), pre-vaccination titer. E), interaction of HIV status and opioid for the log2 whole vaccine fold change.

**Figure S4**

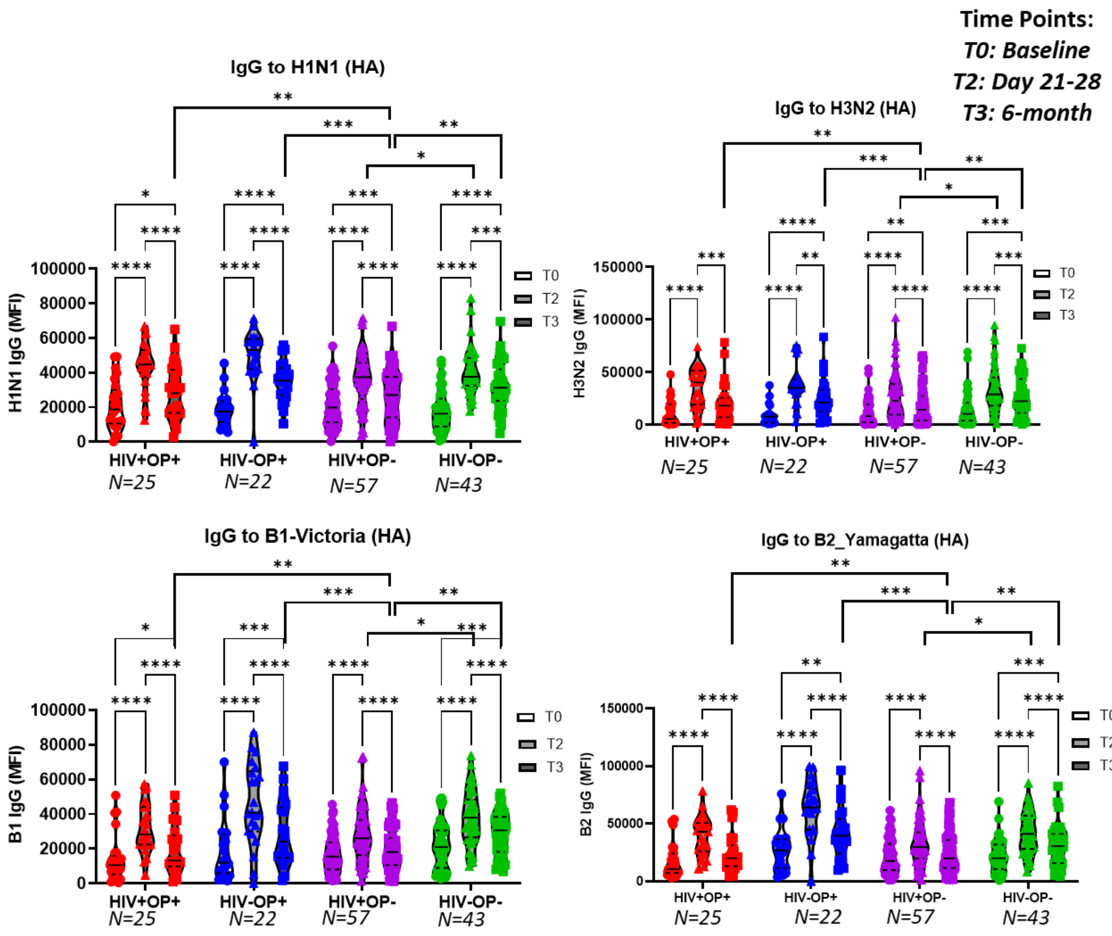


**Figure S4. Antigen-specific HA IgG levels at T0, T2, and T3 after vaccination.** Boxplots show hemagglutinin (HA)-specific IgG levels (MFI) against A/H1N1, A/H3N2, B/Victoria, and B/Yamagata influenza strains at T0 (pre-vaccination), T2 (day 28), and T3 (6 months post-vaccination) in four groups: HIV+OP+ (red; n=25), HIV-OP+ (blue; n=22), HIV+OP- (purple; n=57), and HIV-OP- (green; n=43). Statistical comparisons were performed using mixed-effect models with Geisser-Greenhouse correction, matched values with time points (T0, T1, T2). Tukey's multiple comparisons test, with individual variance computed for each comparison. Adjusted p-values: ****$p<0.0001$, ***$p<0.001$, **$p<0.01$, *$p<0.05$.

**Figure S5**

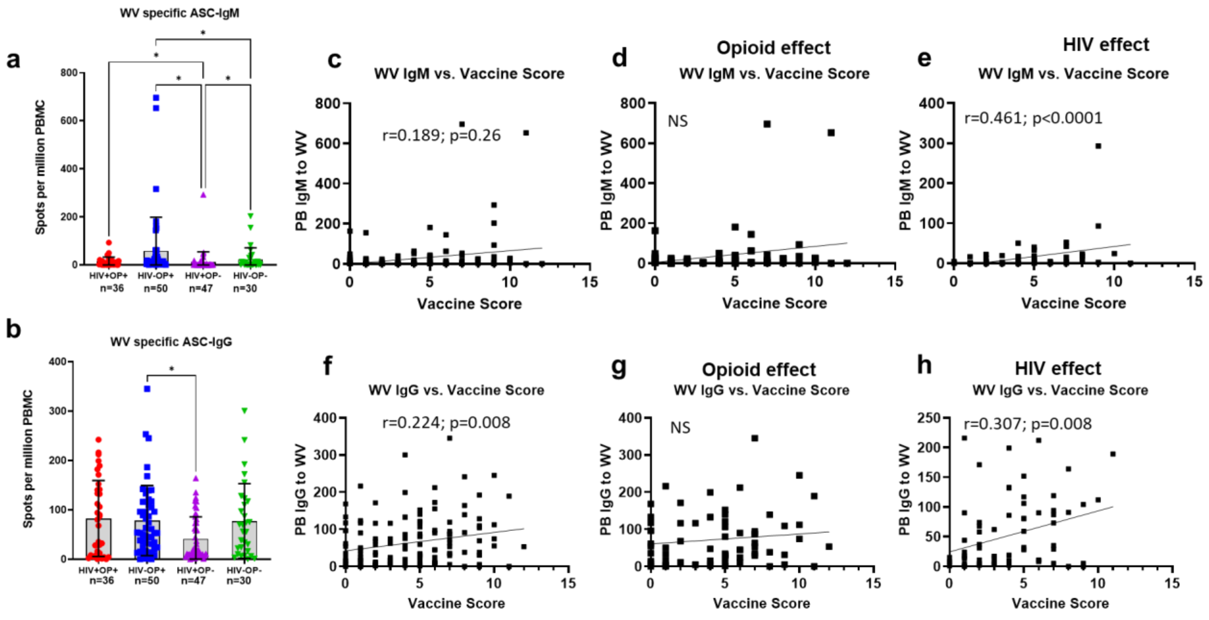


**Figure S5. Lower whole-virus (WV)–specific IgM and IgG plasmablast responses in HIV+OP- at day 7 post-vaccination:** a) Boxplots showing the frequency of whole vaccine-specific IgM antibody-secreting plasmablasts per million PBMCs; b) Boxplots showing whole vaccine-specific IgG plasmablasts per million PBMCs. WV-specific plasmablasts were measured at timepoint T1 (day 7 post-vaccination) using a FluoroSpot assay in a subset of participants (n=36 HIV+OP+; n=50 HIV-OP+; n=47 OP−PWH; n=30 OP−PWoH). Assays were performed on unstimulated PBMCs incubated for 16 hours in WV-coated plates. Statistical significance was assessed using the non-parametric Kruskal-Wallis test: **** = $p < 0.0001$; *** = $p < 0.001$; ** = $p < 0.01$; * = $p < 0.05$.(c-h). Spearman correlation analyses were conducted to assess the association between IgM (c-e) and IgG (f-h) plasmablast responses and vaccine score across all study participants (c, f), as well as within combined opioid (Opioid effect; d,g) and HIV status (HIV effect; e, h) groups.

**Figure S6**

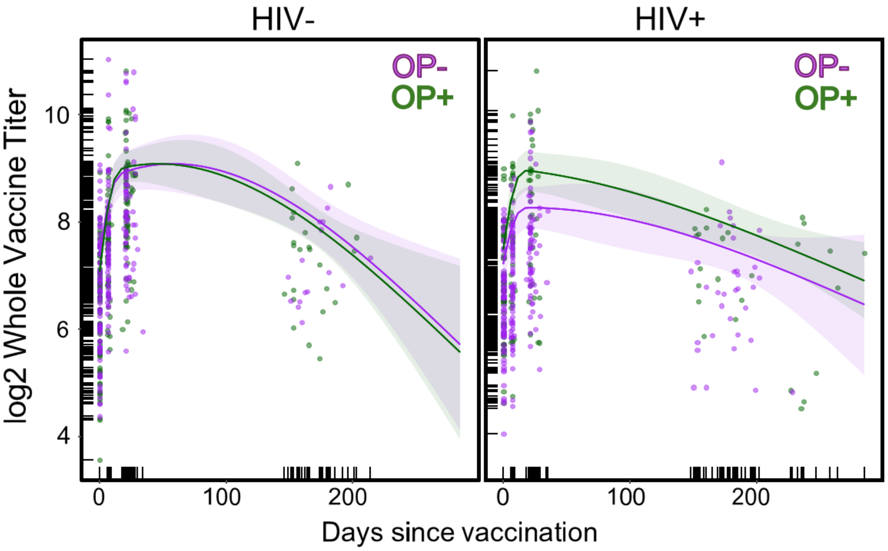


**Figure. S6**. **Results of longitudinal model of log2-transformed whole vaccine antibody titer in relation to HIV status and Opioid use.** 95% confidence bands shown for OP+ (green) and OP- (purple). Curves shown are predicted values from the Natural Cubic Spline regression model and individual data points are the raw values (details in text, Table S3).
